# Influence of Chlorination and Choice of Materials on Fouling in Cooling Water System under Brackish Seawater Conditions

**DOI:** 10.3390/ma9060475

**Published:** 2016-06-15

**Authors:** Pauliina Rajala, Malin Bomberg, Elina Huttunen-Saarivirta, Outi Priha, Mikko Tausa, Leena Carpén

**Affiliations:** 1 VTT Technical Research Centre of Finland, Espoo 02044-VTT, Finland; malin.bomberg@vtt.fi (M.B.); elina.huttunen-saarivirta@vtt.fi (E.H-S.); outi.priha@vtt.fi (O.P.); leena.carpen@vtt.fi (L.C.); 2 Teollisuuden Voima Oyj, Eurajoki 27160, Finland; mikko.tausa@tvo.fi

**Keywords:** biofouling, microbial influenced corrosion, Baltic Sea, biofilm, materials science

## Abstract

Cooling systems remove heat from components and industrial equipment. Water cooling, employing natural waters, is typically used for cooling large industrial facilities, such as power plants, factories or refineries. Due to moderate temperatures, cooling water cycles are susceptible to biofouling, inorganic fouling and scaling, which may reduce heat transfer and enhance corrosion. Hypochlorite treatment or antifouling coatings are used to prevent biological fouling in these systems. In this research, we examine biofouling and materials’ degradation in a brackish seawater environment using a range of test materials, both uncoated and coated. The fouling and corrosion resistance of titanium alloy (Ti-6Al-4V), super austenitic stainless steel (254SMO) and epoxy-coated carbon steel (Intershield Inerta160) were studied in the absence and presence of hypochlorite. Our results demonstrate that biological fouling is intensive in cooling systems using brackish seawater in sub-arctic areas. The microfouling comprised a vast diversity of bacteria, archaea, fungi, algae and protozoa. Chlorination was effective against biological fouling: up to a 10–1000-fold decrease in bacterial and archaeal numbers was detected. Chlorination also changed the diversity of the biofilm-forming community. Nevertheless, our results also suggest that chlorination enhances cracking of the epoxy coating.

## 1. Introduction

Cooling water systems remove heat from components and industrial equipment. Water cooling is typically used for cooling large industrial facilities, such as power plants, chemical factories and petroleum refineries. Cooling systems operate by transferring heat from a heat source, such as a power plant or industrial equipment, to a heat sink, typically water. The most common cooling water systems are once-through systems and open recirculating cooling towers. The former is a common choice, where available water resources are abundant, and thus, they usually use natural water from a lake, river or sea. However, materials are susceptible to fouling in natural waters. Fouling consists of biofouling, corrosion products and precipitation. When seawater is the cooling fluid, the phenomenon is accentuated fundamentally due to the strong corrosive nature of salt water and to its elevated biological activity. Biofouling, inorganic fouling and scaling can reduce heat transfer and enhance corrosion. Corrosion reduces the lifetime of the systems and also further increases scaling and fouling by providing uneven surfaces more favorable for the micro-organisms to attach. Fouling on the other hand reduces the heat exchanging capacity of the system.

Natural water sources contain diverse species of micro-organisms: bacteria, algae and fungi. The interaction between the micro-organisms and the cooling system material surfaces challenges the operational performance of the system by such phenomena as microbial-influenced corrosion (MIC) and microfouling. Microbes can generate conditions that increase corrosion through the alteration of pH and redox potential, the excretion of corrosion-inducing metabolites, direct or indirect enzymatic reduction or oxidation of corrosion products and the formation of biofilms that create corrosive microenvironments [1]. Scaling, where insoluble chemical compounds precipitate on the surfaces, is also possible, although not directly connected with the presence of micro-organisms. Nevertheless, all of these surface processes impair the transfer of heat in the system, thus decreasing the cooling efficiency. Microfouling and inorganic scaling precedes the attachment and growth of macrofouling organisms, such as mussels and macroalgae. Macro-organisms are known to favor surfaces where biofilm is already formed [2,3]. Macrofouling reduces the flow rate of cooling water systems and may reduce also the cooling capacity.

Micro-organisms are typically combated by the use of biocides; in the seawater environment, the common approach is chlorination [4] by, e.g., the addition of hypochlorite compounds [5,6]. However, the proper dosing and timing is a balance between the efficiency of the treatment and the avoidance of the corrosive effect and environmental stress caused by the toxic effect of chlorination treatment. Corrosion may be induced by hypochlorite compounds because they are strong oxidizing agents that easily accelerate the anodic oxidizing reaction and induce the localized attack of passivating alloys, such as stainless steels.

Besides using chemicals, microfouling may be combated by the application of coatings. Special antifouling coatings have been developed [7], with the focus on either surface characteristics, such as hydrophobicity or superhydrophobicity [8], that aim to reduce the contact between the material and its environment, or antimicrobial tendency, *i.e*., the inherent property to influence the microbial counts through interaction between the surface and the micro-organisms.

Seawater is a challenging environment for many metallic materials because of saline content and biological activity. Titanium alloys are commonly-used materials especially in heat exchangers because of their high corrosion resistance, strength and light weight [9]. Steel or coated steel is often used as a pipe material. In this research, we examine biofouling and materials’ degradation in a brackish seawater environment using a range of test materials, both uncoated and coated. Here, the fouling and corrosion resistance of titanium alloy (Ti-6Al-4V), super austenitic stainless steel (254SMO) and epoxy-coated carbon steel (Intershield Inerta160) were studied in the absence and presence of hypochlorite.

This is one of the very few contributions that involves brackish seawater as the studied environment and, thus, improves the understanding of the interactions of micro-organisms typical for such a low-salt water with cooling system material surfaces.

## 2. Results

### 2.1. Biofouling

**Figure 1:**
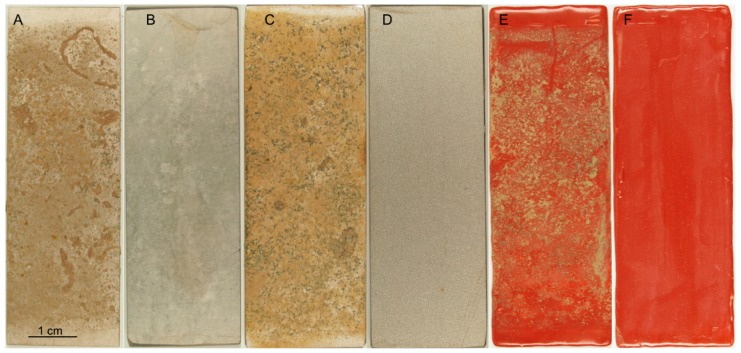
Photographs, showing the specimen surfaces after the three-month test. (**A**,**B**) 254SMO; (**C**,**D**) Ti alloy; (**E**,**F**) Inerta160-coated steel. Non-chlorinated: (**A**,**C**,**E**); chlorinated: (**B**,**D**,**F**).

Fouling was visually evident on all surfaces included in the non-chlorinated system, yet minor fouling had also occurred on the specimens exposed to chlorinated seawater (Figure 1). After one month of exposure, a yellowish deposit could be detected on the specimen surfaces, while after three months of test duration, such a yellowish deposit was accompanied by the presence of individual rod-shaped precipitates. In order to get an insight into the quantity and quality of fouling on the surfaces, molecular biological and microscopy analyses were performed.

#### 2.1.1. Quantification of Micro-Organisms

**Figure 2:**
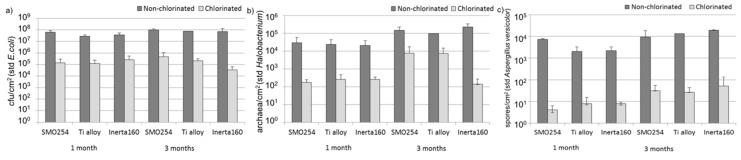
Quantity of (**a**) bacteria; (**b**) archaea (**c**) and fungi, on the coupon surfaces, determined using quantitative PCR. Bars show standard deviations.

The results from quantitative polymerase chain reaction (qPCR) characterizations revealed that there were approximately 10^7^–10^8^ bacteria, 10^4^–10^5^ archaeons and 10^2^–10^4^ fungi per cm^2^ on the coupons, which had been in non-chlorinated seawater for 1–3 months (Figure 2). The material did not have a significant impact on the numbers of attached microbes in the non-chlorinated environment. In the chlorinated environment, a decrease in microbial numbers was clearly seen compared to the non-chlorinated environment. Chlorination decreased the numbers of attached bacteria and archaea by 2–3 and 1–2 orders of magnitude, respectively. Both decreases were statistically significant (*p* ≤ 0.01). Furthermore, in the chlorinated environment after three months of exposure, the number of bacteria and archaea on the surface of Inerta160-coated carbon steel was lower compared to other materials. There were only very few fungi attached to the surfaces, and the decrease of fungi caused by chlorination was not statistically significant. In all cases, the number of biofilm-forming micro-organisms increased on coupons exposed for three months as compared to those being tested for only one month.

#### 2.1.2. Microscopy

**Figure 3:**
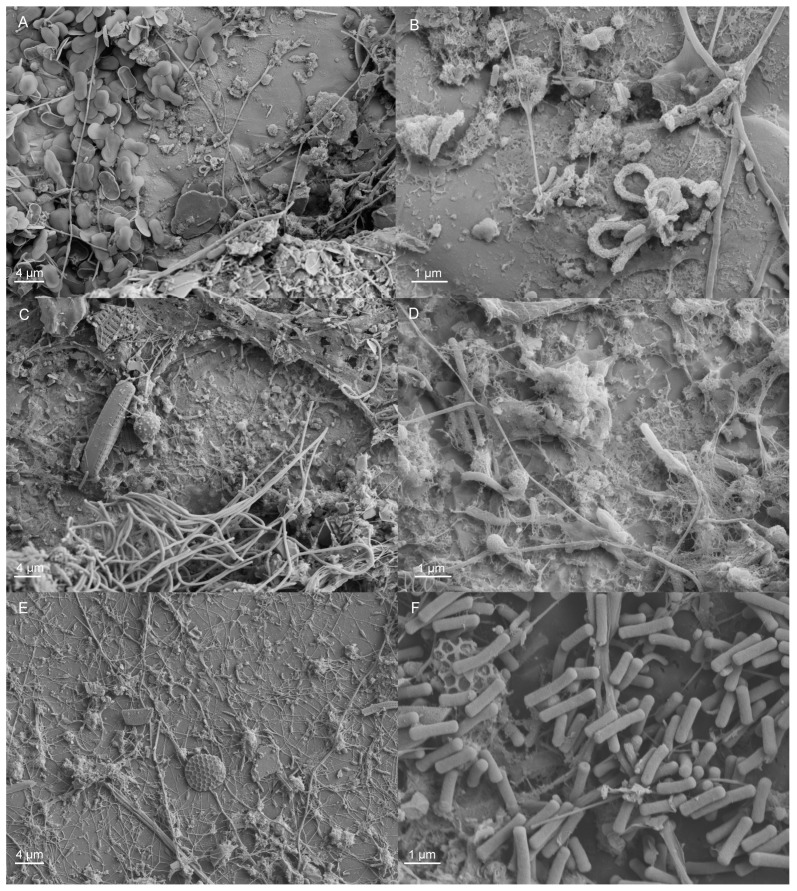
SEM images, showing the surfaces of coupons from the non-chlorinated system after three months of exposure. (**A**,**B**) 254SMO; (**C,D**) Ti alloy; (**E,F**) Inerta160.

**Figure 4:**
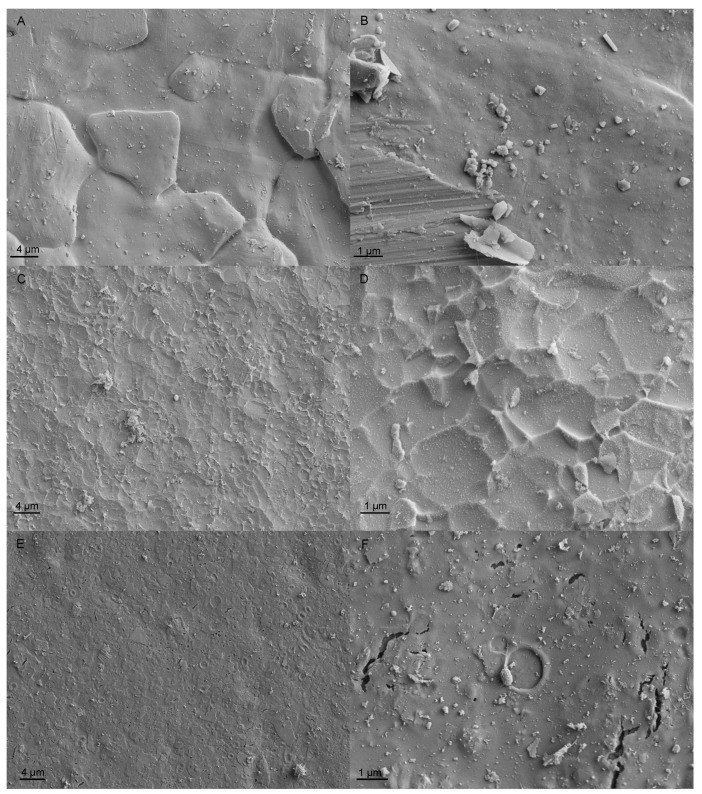
SEM images, showing the surface of coupons from the chlorinated system after three months of exposure. (**A**,**B**) SMO254; (**C**,**D**) Ti alloy; (**E**,**F**) Inerta160.

Examination by scanning electron microscopy (SEM) revealed that, in the non-chlorinated system, the coupon surfaces were abundant with various organisms: algae, including diatoms, protozoa and micro-organisms (Figure 3). A discontinuous and heterogeneous layer of extracellular polymeric substances could also be detected on the surfaces. In the presence of surface irregularities, such as the grain boundaries of the base metal, these were preferentially accommodated by the biofilm. In the chlorinated system, the extent of fouling was clearly reduced as compared to non-chlorinated cases, and very few micro-organisms could be easily distinguished on the specimen surfaces by SEM examinations (Figure 4).

#### 2.1.3. Effect of Chlorination on the Richness and Diversity of the Biofilm

**Figure 5:**
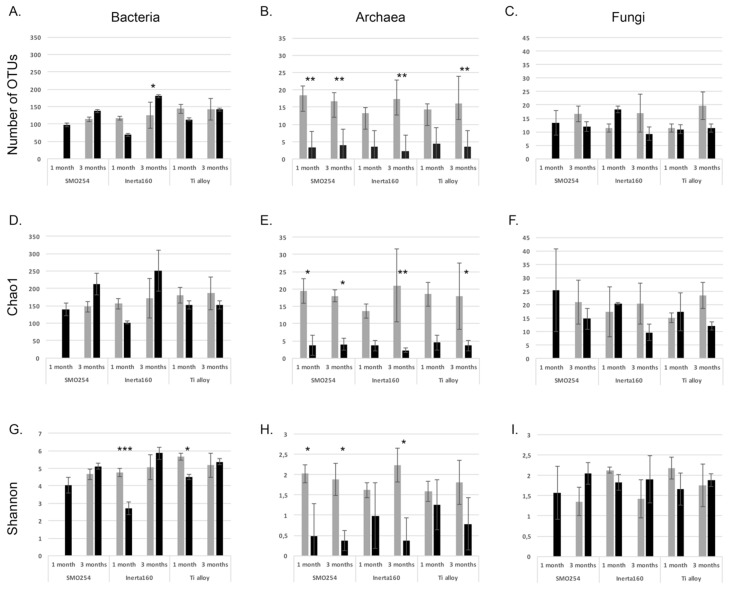
The number of observed Operational Taxonomic Units (OTUs) (**A**–**C**); the number of estimated OTUs according to the Chao1 richness estimators (**D**–**F**); and the Shannon diversity index (**G**–**I**) of the coupon samples. The columns show mean values of three samples based on the normalized number of sequences read per sample, and the error bars indicate the standard deviation. Values calculated for the bacterial, archaeal and fungal data are presented in the left, center and right columns, respectively. Grey columns present non-chlorinated and black columns chlorinated samples. Statistically significantly different sample pairs (non-chlorinated *vs*. chlorinated) are indicated with stars: * *p* < 0.05, ** *p* < 0.005, *** *p* < 0.0005.

The total number of bacterial sequences obtained was 313,947, varying between zero and 15,459 per sample and a mean of 6825 (±4136 standard deviation, STD) sequences per sample. The number of observed Operational Taxonomic Units (OTUs) at the 97% similarity level calculated from the non-rarefied number of sequence reads was between 561 and 2377 OTUs/sample in the non-chlorinated biofilms and 635 and 3416 in the chlorinated biofilms. The number of OTUs estimated by the Chao1 richness index was between 952 and 5752 in the non-chlorinated biofilms and 455 and 3333 in the chlorinated biofilms. The number of bacterial OTUs detected, the Chao1 richness index and the Shannon diversity index were not statistically significantly different in the non-chlorinated treatments compared to the chlorinated ones, except the OTU number between the chlorinated and non-chlorinated three-month exposure on the Inerta160, where the OTU number in the chlorinated treatment was higher than in the non-chlorinated treatment (*p* < 0.03) (Figure 5). In addition, the Shannon diversity indices were also significantly higher in the non-chlorinated one-month exposure with Inerta160 (*p* < 0.0003) and Ti alloy (*p* < 0.05) compared to the chlorinated counterparts.

The total number of archaeal sequences obtained was 972,618, varying between four and 41,687 per sample and a mean of 20,262 (±1955 STD) sequences per sample. The number of observed OTUs at the 97% similarity level calculated from the non-rarefied number of sequence reads was between 1325 and 2739 OTUs/sample in the non-chlorinated biofilms and 57 and 2230 in the chlorinated biofilms. The number of OTUs estimated by the Chao1 richness index was between 2929 and 5752 in the non-chlorinated biofilms and 57 and 5134 in the chlorinated biofilms. The material of the coupons or the exposure time did not affect the archaeal alpha diversity values, but the chlorination strongly affected the alpha diversity of the archaeal communities (Figure 5). The number of archaeal OTUs detected, the Chao1 richness index and the Shannon diversity index were statistically significantly higher in the non-chlorinated treatments compared to the chlorinated ones (*p* < 0.05–0.0009).

The total number of internal transcribed spacer (ITS) sequences obtained was 222,360, varying between zero and 11,711 per sample and a mean of 4941 (±2182 STD) sequences per sample. The number of observed OTUs at the 97% similarity level calculated from the non-rarefied number of sequence reads was between 177 and 374 OTUs/sample in the non-chlorinated biofilms and 69 and 305 in the chlorinated biofilms. The number of fungal ITS OTUs detected, the Chao1 richness index and the Shannon diversity index were not statistically significantly different in the non-chlorinated treatments compared to the chlorinated ones.

#### 2.1.4. Microbial Community Composition

**Figure 6:**
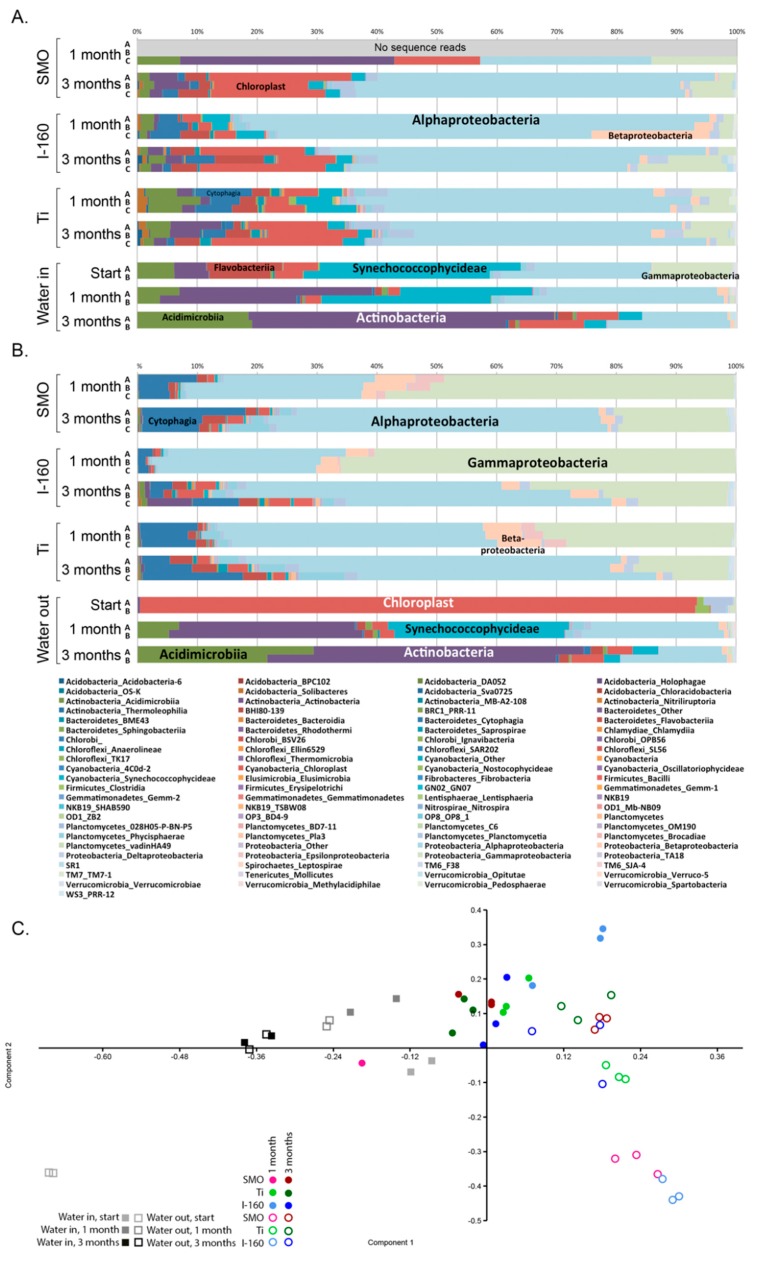
The bacterial community composition of (**A**) the non-chlorinated and (**B**) chlorinated samples; (**C**) a PCA plot based on the bacterial community (family level). In (**C**), open symbols indicate chlorinated and solid symbols non-chlorinated samples. Component 1 explains 39.2% and Component 2 explains 26.2% of the variance, with Actinobacteria, Chloroplasts, Alphaproteobacteria and Epsilonproteobacteria dominating the axis loadings.

The majority of bacterial sequences belonged to Proteobacteria, 42%–95% in the non-chlorinated and 50%–80% in the chlorinated system (Figure 6A,B), of which Alphaproteobacteria formed the largest group. From non-chlorinated biofilm samples, 254SMO 28%–55%, Inerta160 42%–76% and Ti alloy 38%–54% of the bacterial communities belonged to Alphaproteobacteria. Similarly, from chlorinated biofilm, 254SMO 24%–54%, Inerta160 26%–55% and Ti alloy 43%–60% were *Alphaproteobacteria*.

In chlorinated samples, the most abundant alphaproteobacterial species belonged to families Kordiimonadaceae, Rhodobacteraceae and Erythrobacteraceae (Table S1). In addition, on the surfaces of Inerta160, species belonging to the Phyllobacteriaceae family were abundant. The Rhodobacteraceae family was abundant on the surfaces of non-chlorinated samples.

In addition to Alphaproteobacteria, Gammaproteobacteria and Betaproteobacteria were detected on the surfaces. In chlorinated samples, the relative abundance of Gammaproteobacteria was greater than in non-chlorinated samples. The diversity of gammaproteobacterial species was also higher in chlorinated samples. In the chlorinated samples, undetermined Alteromonadaceae, *Oleibacter* and *Pseudoalteromonas* dominated, especially after one month (Table S1). *Pseudoalteromonas* was the most common gammaproteobacterial family in chlorinated 254SMO and Ti alloy samples after one month, forming 20%–48% of the bacterial community, while Alteromonadaceae was the dominating gammaproteobacterial group on Inerta160 samples. In the three-month exposure, Gammaproteobacteria had decreased, and Alphaproteobacteria dominated by Rhodobacteraceae groups had increased. In addition, the chlorinated samples contained Cytophaga in all coupon samples and groups of Betaproteobacteria and Epsilonproteobacteria especially in the one-month samples, which were present at only very low relative abundances in the non-chlorinated samples.

Hetero-organotrophic *Roseivirgra* species belonging to the Bacteroidetes were common in the chlorinated 254SMO and Ti alloy samples contributing 4%–17% of the bacterial sequence reads, but were uncommon in the chlorinated Inerta160 and the non-chlorinated samples. Chloroplasts belonging to Chlorophyta (eukaryotic green algae) were detected in the bacterial 16S rRNA gene sequencing and contributed with high relative abundances especially in the three-month exposure.

In the PCA analysis, there was a clear division between the chlorinated and non-chlorinated samples (Figure 6C).

**Figure 7:**
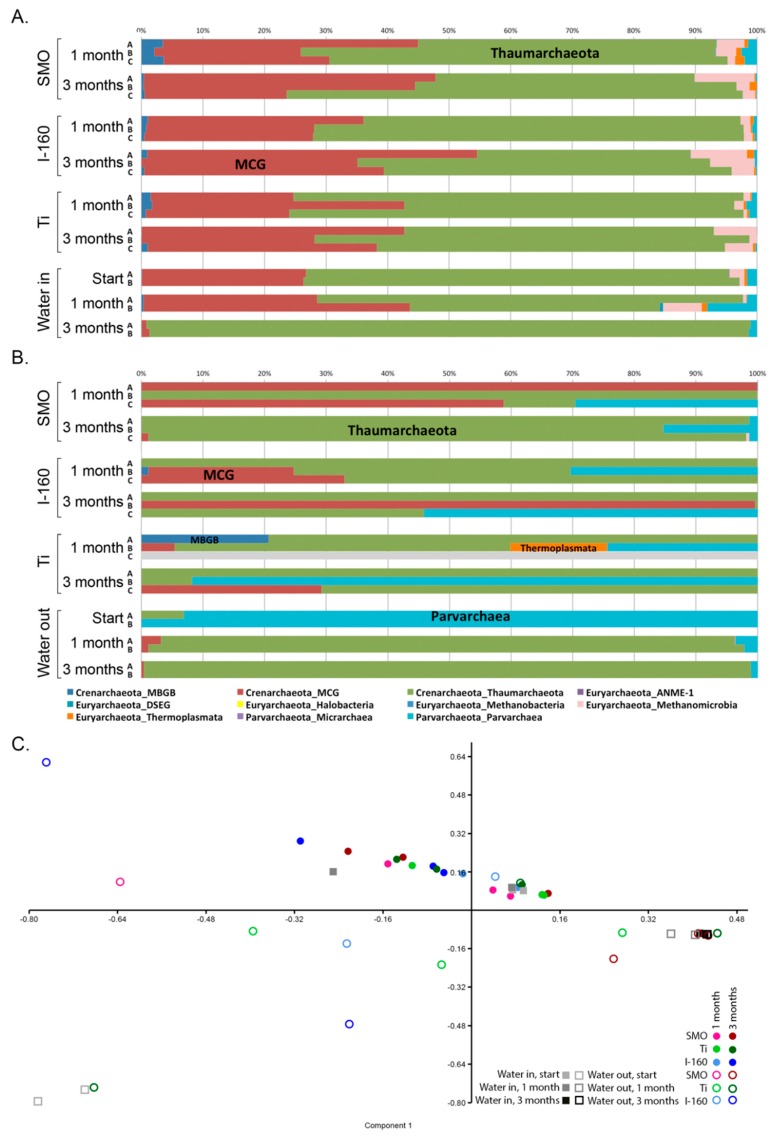
The archaeal community composition of (**A**) the non-chlorinated and (**B**) chlorinated samples; (**C**) a PCA plot based on the archaeal community (family level). In (**C**), open symbols indicate chlorinated and solid symbols non-chlorinated samples. Component 1 explains 67.8% and Component 2 explains 35.1% of the variance, with Thaumarchaeota, the Miscellaneous Crenarchaeotal Group (MCG) and Parvarchaea dominating the axis loadings.

The archaeal community composition did not differ significantly between the different materials or exposure times, but was affected by the chlorination. The majority (85%–100%) of the identified archaeal sequences belonged to the phylum Crenarchaeota (Figure 7A,B). In the non-chlorinated treatments, the majority of the archaea belonged to the thaumarchaeotal *Nitrosopumilus* and the Miscellaneous Crenarchaeotal Group (MCG) linages B10 and pGrfC26 with small inclusions of Methanomicrobia (mostly Methanosarcina), Parvarchaeota and Thermoplasmatales (Figure 7A, Table S2). In the chlorinated treatments, the distribution of different archaeal groups was more heterogeneous. The relative abundance of Parvarchaeota increased especially in the chlorinated water; the MCG archaea were only detected from eight of the 18 coupon samples; and the Methanomicrobia were not detected at all. In addition, from almost all non-chlorinated samples sequence reads belonging to Marine Benthic Group B (MBGB), Methanomicrobia and Thermoplasmatales archaea were identified. In the PCA plot, the chlorinated and non-chlorinated samples mostly separated from each other (Figure 7C).

**Figure 8:**
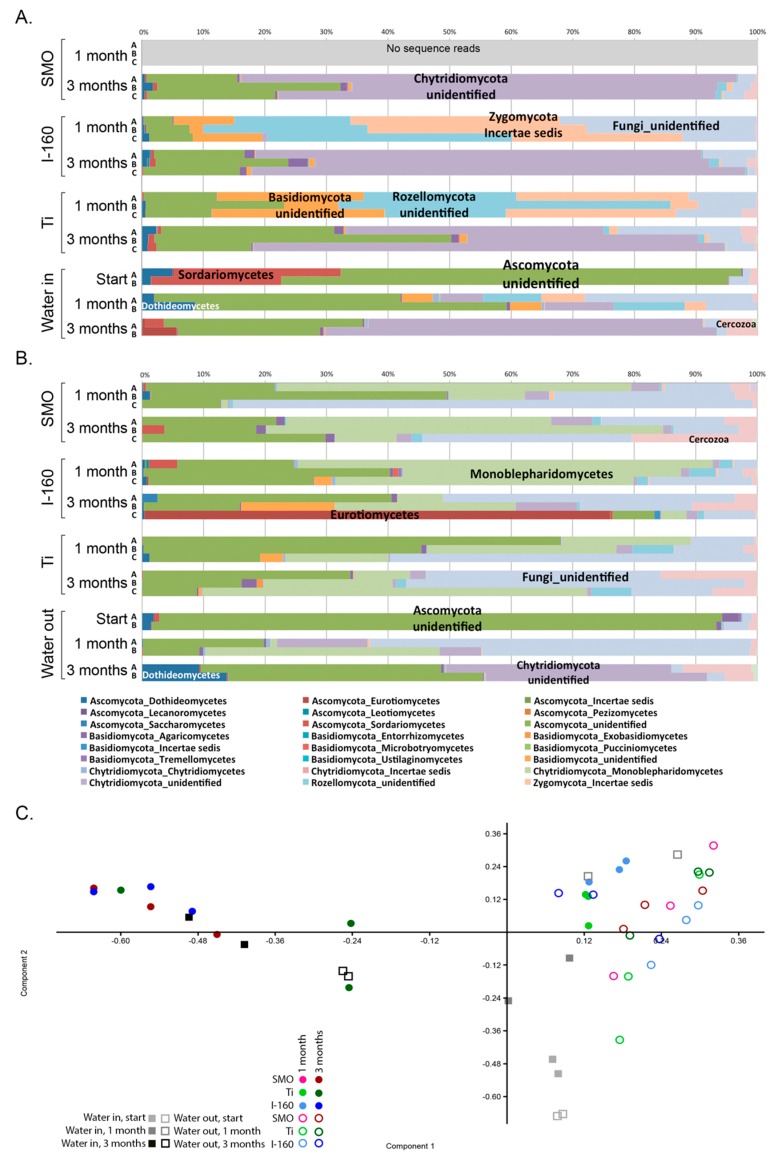
The fungal community composition of (**A**) the non-chlorinated and (**B**) chlorinated samples; (**C**) a PCA plot based on the fungal community (family level). In (**C**), open symbols indicate chlorinated and solid symbols non-chlorinated samples. Component 1 explains 36.7% and Component 2 explains 22.6% of the variance of the variance with Monoblepharidomycetes, unidentified Chytridiomycota and unidentified Fungi determining the loadings of PC1 and unidentified Ascomycota determining the loadings of PC2.

The fungal communities were diverse and differed in composition between the non-chlorinated and chlorinated environments (Figure 8A,B). On the non-chlorinated coupons, Ascomycota, Basidiomycota, Rozellomycota and Zygomycota dominated, but after three months, the fungal communities had changed to consist of mostly Chytridiomycota and Ascomycota. Small amounts of Cercozoa protists were also detected. In the chlorinated environments, the difference between the exposure times was not as clear. The fungal communities in the chlorinated treatments consisted mostly of Ascomycetes and Monoblepharidomycetes with a growing relative abundance of unidentified fungal groups, as well as Cercozoa protists. In the PCA analysis, the one-month Ti alloy and Inerta160 samples grouped closely to the samples from the chlorinated treatments, but all of the three-month samples fell clearly to the other half of the plot into a separate group from the chlorinated samples (Figure 8C).

### 2.2. Inorganic Fouling

To obtain an insight into the nature of inorganic fouling, EDS analyses were conducted on the surfaces. In addition to organic fouling and carbon on the surfaces, also high amounts of silicon, sodium, aluminum, magnesium and chlorine were detected along with oxygen and elements from the alloy. Inorganic scaling particularly on the surfaces of 254SMO was silicon-rich, but the contribution of calcium, aluminum and magnesium was higher and that of carbon much lower than in the non-chlorinated systems (Figure S1). On the surfaces of Ti alloy, only an organic carbon-containing deposit was found at small amounts in addition to elements from the alloy itself (Figure S1).

### 2.3. Materials and Their Performance

The morphology of the epoxy coating (Figure S2) was typical of paints applied by brush, with groove-like features being evident. The coating was continuous, and no pores or cracks could be observed. Cross-sectional examinations revealed a very heterogeneous composite structure of the unexposed coated coupon. The coating matrix contained plenty of fillers, the length scale of which ranged roughly from 100 nm to 10 µm. According to EDS analyses, the most common fillers were oxides of magnesium and silicon, but also niobium, tantalum, molybdenum and barium were occasionally detected (Figure S2). The thickness of the coating was approximately 400–420 µm. Contact angle measurements gave an average of 84 ± 4°; thus, the coating was very slightly hydrophilic (contact angle < 90°).

In the case of alloys, no indications of material degradation could be detected in SEM investigations. However, the grain boundaries in 254SMO became visible during exposure to seawater. In turn, the examination of Inerta160-coated specimens revealed that the coating occasionally contained some cracks or pores after the exposure. In the non-chlorinated system, the size of the cracks after three months was approximately 100–200 nm. The specimens that were exposed for one month only were also studied with respect to such cracks and pores, and there were only a few of them, confirming their appearance as the seawater exposure proceeds. In the chlorinated system, the Inerta160 coating was cracked to a significantly higher degree than in the non-chlorinated system (e.g., Figure 4F). Furthermore, the size of cracks was greater: the crack lengths of up to 2–3 µm were frequently discovered in the specimens exposed for three months.

### 2.4. Seawater

Seawater temperature varied between 17–21 °C for the first 67 days, after which the temperature gradually decreased to 8 °C by the end of the experiment period (Figure S3). Phosphorus, chlorophyll, nitrogen and total organic carbon (TOC) concentrations were determined in the beginning of the experiment, (tot-P 17 µg·L^−1^, a-chlorophyll 2.2 µg·L^−1^, tot-N 310 µg·L^−1^, TOC 4.2–4.5 mg·L^−1^). The microbial numbers in seawater were, on average, 10^6^ bacteria, 10^3^ archaeons and <10^4^ fungi per mL (Table 1). The chlorination decreased the number of microorganisms in the beginning of the chlorination, but later, after the chlorine dose had stabilized, microbial numbers did not differ between chlorinated and non-chlorinated water (Table 1).

The bacterial community in the non-chlorinated seawater at the beginning of the experiment consisted of proteobacteria (33%–38%), cyanobacteria (38%–39%), Actinobacteria (11%–12%) and Bacteroidetes (11%–12%) (Figure 7, Table S1). The chlorination affected the bacterial community at the beginning of the experiment, and the majority of the 16S rRNA gene reads detected belonged to Chlorophyta chloroplasts (94% of the sequence reads). Over time, the bacterial community changed to resemble that of the non-chlorinated input water at one and three months, where the dominating bacterial families belonged to Acidimicrobiia, Actinobacteria, Synechococcophycideae and Alphaproteobacteria after one month and Acidimicrobiia and Actinobacteria after three months. The PCA analysis also set the bacterial communities of the water separately from the biofilm communities (Figure 7C).

The archaea in the non-chlorinated seawater belonged mostly to Thaumarchaeota and MCG archaea, but in the chlorinated water, the archaeal community consisted almost solely of Parvarchaeota. The archaeal communities in both non-chlorinated and chlorinated water changed over time, and while the MCG archaea formed a great part of the non-chlorinated water, the archaeal community in both the chlorinated and non-chlorinated water were dominated by Thaumarchaeota. In the PCA analysis plot, the archaeal non-chlorinated water samples fell close to the non-chlorinated biofilm samples, while the chlorinated outgoing water samples from the beginning of the experiment fall separately from the rest of the samples (Figure 8C).

The fungal community of the non-chlorinated seawater at the beginning of the experiment consisted mostly of unidentified Ascomycota and of Sordariomycetes fungi, and the fungal community became more diverse over time. The relative abundance of Sordariomycetes decreased and was replaced by Basidiomycota, Chytridiomycota, Zygomycota and Rozellomycota. During the final sampling, Chytridiomycota was the dominating fungal group, and the relative abundance of Cercozoa protists had increased. In the chlorinated water, the Sordariomycetes almost disappeared due to the chlorination already at the beginning of the experiment, and unidentified Ascomycota was the dominating fungal group. At the one-month sampling point, the proportion of unidentified fungi had increased to over 50% of the community, and the Ascomycota had dramatically decreased. At the end of the experiment, the Ascomycota had recovered and formed again approximately 40% of the fungal community, and the relative abundance of *Chytridiomycota* and *Dothideomycetes* had increased. The Cercozoa protists were also well represented in the chlorinated outgoing water at the end of the experiment period (Figure 8A,B). The PCA analysis placed the non-chlorinated water samples from the beginning and one-month time point separate from the rest of the samples and the corresponding chlorinated water samples close to the biofilm samples from the chlorinated and the non-chlorinated one-month samples. After three months, the non-chlorinated water samples fell with the non-chlorinated three-month biofilm samples (Figure 8C).

## 3. Discussion

Cooling water systems utilizing natural waters are very demanding applications with respect to the reliable long-term performance of materials and coatings. The results obtained here demonstrated that biological fouling is intensive in cooling systems using brackish seawater in sub-arctic areas during the summer period. The microfouling comprised a vast diversity of bacteria, archaea, fungi, algae and protozoa. Our results demonstrate that chlorination was effective against biological fouling; up to a 10–1000-fold decrease in bacterial and archaeal number was detected. When interpreting the results from the quantitative PCR assay, it has to be borne in mind that the gene copy numbers vary in different microbial species. Thus, the results do not give absolute numbers, but still give an overview estimate of the trend and can be well used for comparing different samples. The coating, Inerta160, combined with chlorination further improved the antifouling effect. However, in non-chlorinated environments, the material selection did not have any significant effect on biofouling.

In addition to reducing the number of micro-organisms, the chlorination also had an effect on species diversity attached to surfaces compared to the non-chlorinated environment. Principal component analysis (PCA) was utilized to visualize the difference in sample clustering according to the species detected. In the PCA analysis, there was a clear division of biofouling species between the chlorinated and non-chlorinated environment. Alphaproteobacteria dominated in the biofilm in the non-chlorinated environment regardless of the material, and the Rhodobacteraceae family was abundant on the surfaces of non-chlorinated samples. The relative abundance of alphaproteobacteria increased with longer exposure time, and the biofilm became thicker over time. In chlorinated samples, the majority of the alphaproteobacteria belonged to the families Kordiimonadaceae, Rhodobacteraceae and Erythrobacteraceae. Many bacteria belonging to Rhodobacteraceae are phototrophic iron oxidizing [10], whereas the Kordiimonadaceae family is often detected from marine environments where they degrade hydrocarbons [11]. Members of the Erythrobacteraceae family are also commonly detected from marine systems and may oxidize organic carbon compounds [12]. Erythrobacteraceae may also oxidize manganese, a process that has been linked to localized corrosion of stainless steels [13]. Bacteria belonging to both Rhodobacteraceae and Erythrobacteraceae have previously been identified as forming biofilms on copper-based antifouling coating in marine systems [14]. In fact, Erythrobacteraceae bacteria had 10–100-times higher relative abundances in the biofilms of the chlorinated systems compared to the non-chlorinated ones (Table S2).

Gammaproteobacterial *Pseudoalteromonas* species were tolerant to chlorination and formed the majority of the biofilm in the chlorinated environment. Some *Pseudoalteromonas* species produce compounds that have antibacterial, algicidal, antifungal or antiviral activity and may thus have a beneficial position when competing for space in biofilm [15]. On the other hand, certain *Pseudoalteromonas* species attract other organisms to surfaces [16]. *Pseudoalteromonas* species originating from marine water have been shown to cause corrosion of duplex stainless steel where the bacteria induced local corrosion by decreasing the chromium content under the biofilm and accumulated chloride ions on the metal surfaces [17]. The accumulation of chloride, as well as the apparent enrichment of *Pseudoalteromonas* in the biofilms of the 254SMO and Ti alloy coupons of our chlorinated systems indicate that this bacterium may not be susceptible to the chlorination treatment. The relative abundance of Bacteroidetes in biofilm in a chlorinated environment was also higher compared to the non-chlorinated system and the majority of these resembled hetero-organotrophic *Roseivigra* species. After prolonged exposure time, the composition of the biofilms became more uniform on the surface of different materials.

Most studies concentrate mainly on the bacterial fraction of biofouling, and thus, the role of the archaeal community in biofouling is not well known. Archaeal diversity on surfaces was significantly affected by chlorination, but although a small part of the archaeal community consisted of hydrogenotrophic methanogenic archaea, in both the chlorinated and non-chlorinated treatments, Crenarchaeotal lineages (Thaumarchaeota and MCG) dominated. Our results agree with previous studies on the MIC of oil pipes [18,19] or carbon steel in marine environments [20], where the archaeal counterparts causing corrosion have belonged to different hydrogenotrophic methanogenic species. However, to the best of our knowledge, Crenarchaeotal (Thaumarchaeota and MCG) or Parvarchaeota species have not been associated with the MIC of stainless steel or titanium, consistent with our observations. It is very possible that these archaea do not primarily cause corrosion, but they may facilitate MIC and biofilm formation by capturing inorganic carbon into the organic form and recycle nitrogen compounds (ammonia) from decomposing senescent biofilms for the benefit of the other microbial community [21,22] members that cause MIC. In addition, the MCG archaea have been shown to have genes encoding extra-cellular protein-degrading enzymes [23], which would assist in the degradation of organic matter, such as old biofilms, releasing ammonia for the Thaumarchaeota, which may then produce nitrite and nitrate for the nitrite- and nitrate-reducing bacteria.

Fungi were generally detected at lower concentrations than the bacteria and archaea, but the fungal communities on the specimen surfaces and in the water were surprisingly diverse. Despite their low abundances, fungi may play a great role in the formation of biofilms, because through their metabolism, the fungi consume oxygen efficiently, thus providing anoxic microhabitats for, e.g., anaerobic sulfate reducers or the methanogenic archaea to operate. Ascomycetes fungi, such as *Aspergillus niger*, have been reported to cause corrosion on magnesium alloy in artificial seawater [24]. Diverse fungi also caused severe corrosion damage to storage tanks and transporting pipelines for oil [25]. Ascomycetes oxidize manganese [26], and fungi secrete different types of exudates, such as organic acids and enzymes, which may locally lower the pH and induce localized corrosion and cracking [27]. In agreement with our results, Pereira *et al*. [28] also found a variable effect of chlorination on the inhibition of Ascomycetes fungi, and Ascomycetes were detected in both the chlorinated and non-chlorinated systems. Clearly, species belonging to Chytridiomycota, Rozellomycota and Zygomycota were more susceptible to chlorination than the Ascomycota, while species of the Monoblepharidomycetes were detected only in the chlorinated treatments.

Chlorophyta chloroplasts were relatively abundantly detected in the bacterial 16S rRNA gene sequence data, especially in the non-chlorinated systems, indicating the presence of eukaryotic green algae. Chlorophyta are common inhabitants of fouling biofilms on different surfaces (e.g., [29,30]). Hydrogenase-containing Chlorophyta are able to use cathodic hydrogen and may, under acidic conditions, contribute to corrosion events [31]. Other non-fungal eukaryotes detected were the Cercozoa protists, which were especially abundant in the chlorinated systems. These protozoans are also common in biofilms and may serve as hosts for pathogenic bacteria, such as Legionella [32].

Besides biological fouling, also the extent and nature of organic and inorganic fouling changed due to chlorination. Because of chlorination, an overall decrease in the extent of fouling was detected. Carbon was detected in all analyses, indicating that the presence of biofilm also contributed to the results, but it is clear that much of the information also originated from inorganic deposits on the surface. The results indicated that in non-chlorinated systems, the biofilm development facilitated inorganic scaling, particularly SiO_2_. Indeed, e.g., SiO_2_ is a common inorganic constituent in scales developed in seawater environments [33,34,35], with the biofilm formation known to further enhance its deposition on the surfaces [33]. This is also the case here, because carbon and silicon were systematically detected in the same analyses. In the chlorinated systems, the contribution of “softer” inorganic constituents, such as Ca, Mg and Al, increased, and SiO_2_ that forms hard and tenacious deposits correspondingly decreased, thus demonstrating the viability of chlorination in the scale modification. This is an important aspect with respect to long-term performance and maintenance of seawater cooling systems, because the removal of SiO_2_ scales may be challenging due to their high hardness [35].

The composition of the coating was studied prior to exposure and was detected to contain heterogeneous composite structure, having abundant fillers. The most common fillers were oxides of magnesium and silicon with niobium, tantalum, molybdenum and barium. It is possible that barium is included in the coating as barium sulfate, a common anti-fouling agent used due to its favorable price [36]. In EDS spectra, the peaks of molybdenum and sulfur overlap, hiding the sulfur in the results. Furthermore quartz, SiO_2_, is a frequently-used low-price filler that improves the wear resistance of the coating. Another important practical finding is the enhanced cracking of the epoxy coating Inerta160 in the chlorinated system. It is expected that the cracks did not reach the substrate during the three-month exposure period, because no corrosion products of steel were detected in the epoxy coating. However, the operation of epoxy coatings relies on the barrier effect, *i.e.*, the non-conducting layer physically insulating the substrate (here steel) from the environment, which was challenged by chlorination. Earlier, UV radiation [37] and thermal oxidation [38] have been linked with the cracking of epoxy-based coatings.

The overall experiment period was short, three months at the longest, and lasted for only one warm period (summer season) when pulse chlorination is utilized to prevent biofouling. The chlorination was ended for the colder winter season, the same time as the experiment period ended. The rate of biofilm formation is known to correlate with sea water temperature, which changes with the season [39]. This is especially important in sub-arctic areas, such as the Baltic Sea area, where this study was conducted, where the temperatures change drastically between, e.g., summer and winter periods. To obtain reliable results of the effects of the fouling and chlorination on materials’ performance, experiments lasting for several chlorination periods are needed. Nevertheless, during this three-month experiment period, it was already demonstrated that chlorination has a clear effect on the fouling and species composition of the biofilms developing on the surfaces.

## 4. Materials and Methods

### 4.1. Materials

Two of the materials were uncoated: super austenitic stainless steel 254SMO and titanium-based alloy Ti-6Al-4V. The composition of stainless steel 254SMO was 0.01 wt% C, 0.20% N, 20.0% Cr, 18.0% Ni, 6.1% Mo, 0.7% Cu and Fe (balance). For Ti-6Al-4V, the composition was 6.0 wt% Al, 4.0% V, ≤0.25% Fe, ≤0.2% and Ti (balance). Test materials also included epoxy-coated carbon steel. Red epoxy was an abrasion-resistant epoxy Intershield 163 Inerta160 (International Marine Coatings, Gateshead, UK) intended for corrosion protection applications in the underwater areas of ice-going vessels. The coatings were applied as recommended by the manufacturer. In each case, the coupon size was 25 × 75 mm.

Coupons were cleaned with FreeBact-20 (AquaFix, Saltsjöbaden, Sweden) and sterile MQ water and air-dried. Each biofouling cell contained 8 coupons of each material. Three parallel coupons were used for material analyses, three coupons for molecular biological analyses and two for microscopy analyses. The coupons were photographed prior to the experiment.

### 4.2. Experiment Setup

The test materials were placed in a biofouling cell in the cooling water system of a nuclear power plant located in the western coast of Finland. Two cells were installed in the cooling water cycle before the chlorination point and two cells after the chlorination point. The water flow through the biofouling cell was set at 0.01 m·s^−1^ using rotameters. The chlorination of the cooling water system was started in July 2015 and lasted 106 days. For chlorination, 15% of Na-hypochlorite was used with a final target concentration 3 mg·L^−1^. During the first 30 days, chlorination was applied for two hours with 10 h brakes between treatments and, after that, for one hour with 11 h breaks between. The experimental period started a week after chlorination treatment was initiated. One cell before the chlorination point and one cell after it were removed after 1 month (32 days) of exposure. The remaining two cells were removed after 3 months (90 days) of exposure. After the removal of the biofouling cells from the water cycle, the cells were sealed to retain the water inside them. The cells were dismantled aseptically in the laboratory.

### 4.3. Surface Characterization

Coupons for corrosion studies were immersed in 96% ethanol, air-dried and placed in glass desiccators. All coupons for corrosion analyses were photographed and examined with a stereomicroscope to verify the surface condition. The surface deposits of selected coupons were analyzed with a scanning electron microscopy (SEM) and energy-dispersive spectroscopy (EDS). Furthermore, the as-received Inerta160-coated steel specimens were subjected to investigations by SEM-EDS to disclose the original coating structure in the surface and cross-section. To enable the investigations, a thin layer of gold was sputtered on the coated specimens to make them electrically conductive. Field-emission (FE) SEM Zeiss ULTRAplus equipped with a Noran EDS system was employed for the investigations. Surface characteristics (hydrophobicity/hydrophilicity) of the Inerta160 coating were studied using an unexposed specimen by contact angle measurements.

To visualize the biofilm formed on the surface, selected samples were fixed in phosphate (0.1 M, pH 7.2), buffered with 2.5% glutaraldehyde for 2 h and dehydrated with ethanol series followed by final drying in hexamethyldisilazane. The specimens were coated with Au/Pd (10–15 nm) and examined with a Zeiss SIGMA VP field-emission scanning microscope (FESEM; Carl Zeiss SMT GmBH, Oberkochen, Germany).

### 4.4. DNA Extraction

Coupons for microbiological analyses were subjected to biofilm extraction immediately after they were removed from the biofouling cell. The biomass from the coupon surfaces was removed by sonicating the coupons for 10 min (Bransonic 2210-DTH, Branson Ultrasonics, Danbury, CT, USA, 47 kHz, 70W) and thereafter vortexing for 1 min. The biomass suspensions were filtered into Sterivex-GP 0.22-µm filter units (Millipore, Billerica, MA, USA) and stored frozen (−80 °C) until DNA was extracted. Non-chlorinated and chlorinated water samples were filtered at the beginning of the experiment and at both sampling times as two 500-mL replicates to Corning PES (polyethersulfone) 0.22-µm filter units (Corning, New York, NY, USA), which were cut out with a sterile scalpel and stored frozen (−80 °C). Subsequently, the Sterivex filter units were aseptically broken with a hammer, and the Sterivex and Corning filters were cut into pieces with a sterile scalpel and placed into the lysing tube of the DNA extraction kit with sterile forceps. DNA was extracted using the Fast DNA Spin Kit for Soil (MP Biomedicals, Santa Ana, CA, USA) according to the manufacturer’s instructions, with the modification that the cells were homogenized in a FastPrep-24 instrument (MP Biomedicals, Santa Ana, CA, USA) at 6 m·s^−1^ for 3 min.

### 4.5. Quantitative PCR

The bacterial, archaeal and fungal biomass on the surface of the samples and in seawater was evaluated with quantitative PCR (qPCR) using the LightCycler 480 instrument (Roche Diagnostics, Basel, Switzerland). The DNA concentration of all samples was adjusted to ≤10 ng·μL^−1^ prior to qPCR in order to avoid PCR inhibition. For bacteria, an approximately 200-bp fragment of the 16S rRNA gene was amplified with primers 358F (5′-CCT ACG GGA GGC AGC AG-3′) and 534R (5′-ATT ACC GCG GCT GCT GG-3′) [40] and detected with SYBR green-based detection for double-stranded DNA. The amplification was done in a 10-µL reaction volume with KAPA Sybr Fast qPCR Master mix optimized for Roche LightCycler 480 (KAPA Biosystems, Wilmington, MA, USA), 150 nM of each primer and 1 µL of sample DNA. The amplification reaction consisted of initial denaturation at 95 °C for 15 min, 45 cycles with 10 s at 95 °C, 35 s at 57 °C and 30 s at 72 °C, a final elongation of 3 min at 72 °C and a melting curve analysis. As an external standard, a dilution series (100–10^8^ cfu/µL) of *Escherichia coli* VTT E-90418 genomic DNA was used. For archaea, an approximately 400-bp fragment of the 16S rRNA gene was amplified with primers A344F (5′-ACG GGG TGC AGC AGG CGC GA-3′) [41] and A744R (5′-CCC GGG TAT CTA ATC C-3’) modified from 744RA [42] and detected with SYBR green. The amplification was done in a 10-µL reaction volume with KAPA Sybr Fast qPCR Master mix optimized for Roche LightCycler 480 (KAPA Biosystems, Woburn, MA, USA), 300 nM of each primer and 1 µL of template DNA. The amplification reaction consisted of initial denaturation at 95 °C for 15 min, 45 cycles with 10 s at 95 °C, 35 s at 56 °C and 30 s at 72 °C, final elongation of 3 min at 72 °C and a melting curve analysis. As an external standard, a dilution series of *Halobacterium salinarum* VTT E-103154^T^ genomic DNA was used (100–10^7^ cells/µL). Fungal biomass was detected with TaqMan probe assay using ITS (internal transcribed spacer) as a target. Primers 5.8F1 (5′-AAC TTT CAA CAA CGG ATC TCT TGG-3′) and 5.8R1 (5′-GCG TTC AAA GAC TCG ATG ATT CAC-3′) and probe 5.8P1 (5′-CAT CGA TGA AGA ACG CAG CGA AAT GC-3’) were used [43]. The amplification was done in 10 µL reaction volume with KAPA PROBE FAST qPCR Kit (KAPA Biosystems, Woburn, MA, USA), 500 nM of each primer, 200 nM of probe, and 1 µL of template DNA. The amplification reaction consisted of enzyme activation at 95 °C for 3 min and 40 cycles with 10 s at 95 °C (denaturation), 30 s at 62 °C (annealing) and 1 s at 72 °C (elongation). As an external standard, a dilution series of *Aspergillus versicolor* VTT D-96667 genomic DNA was used (10–10^5^ spores/µL).

### 4.6. Amplicon Library

The amplification libraries for high throughput sequencing with Ion Torrent PGM were prepared by PCR from the DNA samples. Bacterial 16S genes were amplified with primers S-D-Bact-0341-b-S-17/S-D-Bact-0785-a-A-21 [44], targeting the variable region V3-V4 of the 16S rDNA gene, S-D-Arch-0349-a-S-17/S-D-Arch-0787-a-A-20 [44], archaeal 16S genes with primers S-D-Arch-0349-a-S-17/S-D-Arch-0787-a-A-20 [44], targeting the V4 region of the gene and fungal internal transcribed spacer (ITS) gene markers with primer pair ITS1 and 58A2R targeting the fungal ITS1 region [45,46]. PCR amplification was performed in parallel 25-μL reactions for every sample containing 1× MyTaq™ Red Mix (Bioline, London, UK), 20 pmol of each primer, up to 25 μL molecular-biology-grade water (Sigma, St. Louis, MO, USA) and 2 μL of template. The PCR program consisted of an initial denaturation step at 95 °C for 3 min, 35 cycles for bacteria and fungi and 40 cycles for archaea of 15 s at 95 °C, 15 s at 50 °C and 15 s at 72 °C. A final elongation step of 30 s was performed at 72 °C. The PCR products were verified with agarose gel electrophoresis. Amplicons were sent to Ion Torrent sequencing with PGM equipment (Bioser, Oulu, Finland), and amplicons were purified before sequencing at Bioser.

### 4.7. Sequence Processing and Analysis

The sequence reads obtained from Ion Torrent sequencing were subjected to quality control using the QIIME-software Version 1.8 [47] using a minimum quality score of 20, minimum and maximum sequence length of 200 bp and 600 bp, respectively, maximum primer mismatch of 2 nucleotides (nt) and maximum homopolymer stretches of 8 nt. Adapters, barcodes and primers were removed from the sequence reads, and chimeric sequence reads were removed from the dataset with the USEARCH-algorithm [48] by *de novo* detection and through similarity searches against the Greengenes reference dataset (Version gg_13_8) [49] with bacterial and archaeal sequences, as well as the UNITE (User-friendly Nordic ITS Ectomycorrhiza Database) reference dataset (Version sh_taxonomy_qiime_ver7_97_s_31.01.2016) [50] with fungal sequences.

The sequences were grouped into Operational Taxonomic Units (OTUs), following the open-reference OTU-picking protocol of QIIME-software. First, all reads that failed to hit the Greengenes or UNITE reference database with a minimum of a 60% identity threshold were discarded as sequencing errors. Subsequently, closed-reference OTUs were picked at 97% clustering identity against the Greengenes or UNITE database, and *de novo* OTUs were picked from a randomly subsampled sequence subset that failed the closed-reference OTU-picking stage. Next, singleton OTUs, *i.e*., OTUs that were represented by a single sequence, were filtered from the dataset. Finally, taxonomy from the domain level to the species level was assigned to OTUs via representative OTU sequences with the Ribosomal Database Project (RDP) classifier algorithm at a minimum of 80% confidence [51] with bacterial and archaeal sequences. With fungal ITS sequences, taxonomic assignments were made by the Basic Local Alignment Search Tool (BLAST) algorithm with a maximum E-value of 0.001 [52]. Unassigned and unidentified sequences were filtered from the datasets before alpha diversity estimations. Alpha diversity indices were calculated on randomly subsampled and filtered datasets containing 1243, 1674 and 1624 sequences for ITS, bacteria and archaea, respectively.

The sequences were deposited in the European Nucleotide Archive (ENA, https://www.ebi.ac.uk/) under accession number PRJEB14388.

### 4.8. Statistics Analyses

Significant differences of the qPCR means were tested with variance analysis using sampling time, surface material and chlorination as fixed factors and the subsequent Tukey’s *post hoc* test using IBM SPSS Statistics Version 21 software. The results were log-transformed to fulfil the assumptions of variance analysis.

Differences in the number of OTUs detected from the different biofilm samples, the Chao1 richness estimates and Shannon indices were tested based on normalized sequence data using One-way ANOVA in the PAST3 program [52]. We tested significant differences of the mean between chlorinated and non-chlorinated samples using Levine’s test for the homogeneity of variance, Kruskal–Wallis and Tukey’s Q test. The difference in community composition between all samples was tested with principal component analysis of 100 bootstrap repeats on the taxonomic relative abundance data using the PAST3 program.

## 5. Conclusions

The present work demonstrates the powerfulness of molecular biological tools in studying biofouling, especially microfouling in cooling water environments. Biofilm formation in the non-chlorinated system was intensive, but also in the chlorinated system, a biofilm consisting of bacteria, archaea, fungi, algae and protozoa was detected. According to the results presented here, the chlorination reduces microfouling in brackish water by 10–1000-fold, but also alters the structure of the remaining community forming the biofilm on surfaces and the composition of inorganic scaling. In addition, the choice of material had an effect on the biofilm-forming community in the beginning, but prolonged exposure time unified the composition of the biofilm-forming community between different materials. Multiple potentially corrosion-inducing species, e.g., manganese oxidizing, sulfate reducing and iron oxidizing bacteria, were detected on the surfaces of all materials. In addition to having a reducing and species selecting influence on biofilm formation, the chlorination also deteriorated the epoxy coating already during one month of exposure. On the other hand, coating combined with chlorination further reduced the microfouling on the surfaces compared to metallic surfaces.

## Figures and Tables

**Table 1:** Microbial numbers in sea water, as determined by quantitative PCR, using *Escherichia coli*, *Halobacterium salinarum* and *Aspergillus versicolor* as external standards.

	Bacteria, cfu/mL		Archaeons, cells/mL		Fungi, spores/mL	
	Non-Chlorinated	Chlorinated	Non-Chlorinated	Chlorinated	Non-Chlorinated	Chlorinated
beginning	7.7 × 10^6^	1.8 × 10^3^	3.2 × 10^3^	<1	2300	3
1 month	8.3 × 10^6^	5.6 × 10^6^	8.8 × 10^3^	6.8 × 10^3^	500	380
3 months	5.8 × 10^6^	8.8 × 10^6^	1.3 × 10^4^	6.8 × 10^3^	360	710

## Supplementary Materials

The following are available online at www.mdpi.com/1996-1944/9/6/475/s1. Figure S1: EDS spectra for coupons. (a) 254SMO chlorinated; (b) Ti alloy chlorinated; (c) 254SMO non-chlorinated system; (e) Ti alloy non-chlorinated system. Figure S2: SEM images. (a) Surface; (b) cross-section; (c–e) EDS spectra for particles included in the Inerta160 coating. Figure S3: Temperature (°C) of the seawater during the experiment. Table S1: Diversity and sequence stats. Table S2: Bacteria, relative abundance and genus level. Table S3: Archaea, relative abundance and genus level. Table S4: Fungi, relative abundance and genus level.

## Abbreviations

The following abbreviations are used in this manuscript:

ANOVA	Analysis of variance
BLAST	Basic Local Alignment Search Tool
bp	Base pair (in DNA)
DNA	Deoxyribonucleic acid
FE-SEM	Field emission scanning electron microscopy
EDS	Energy-dispersive spectroscopy
ITS	fungal internal transcribed spacer
MCG	Miscellaneous crenarchaeotal group
OTU	Operational taxonomic unit
PCA	Principal component analysis
PCR	Polymerase chain reaction
STD	Standard deviation
Ti	in this paper, Ti-6Al-4V
TOC	Total organic carbon

## References

[1] Fleming H.C., Heitz E., Fleming H.C., Sand W. (1996). Microbially Influenced Corrosion of Materials.

[2] Sawall Y., Richter C., Ramette A. (2012). Effects of eutrophication, seasonality and macrofouling on the diversity of bacterial biofilms in equatorial coral reefs. PLoS ONE.

[3] Salta M., Wharton J.A., Blache Y., Stokes K.R., Briand J.F. (2013). Marine biofilms on artificial surfaces: Structure and dynamics. Environ. Microbiol..

[4] Cristiani P., Perboni G., Debenedetti A. (2008). Effect of chlorination on the corrosion of Cu/Ni 70/30 condenser tubing. Electrochim. Acta.

[5] Cristiani P., Perboni P. (2014). Antifouling strategies and corrosion control in cooling circuits. Bioelectrochemistry.

[6] Murthy P.S., Venkatesan R., Nair K.V.K., Inbakandan D., Jahan S.S., Peter D.M., Ravindan M. (2005). Evaluation of sodium hypochlorite for fouling control in plate heat exchangers for seawater application. Int. Biodeter. Biodegrad..

[7] Yebra D.M., Kiil S., Dam-Johansen K. (2004). Antifouling technology—Past, present and future steps towards efficient and environmentally friendly antifouling coatings. Prog. Org. Coat..

[8] Ferrari M., Benedetti A. (2015). Superhydrophobic surfaces for applications in seawater. Adv. Coll. Interf. Sci..

[9] Gurrappa I. (2003). Characterization of titanium alloy Ti-6Al-4V for chemical, marine and industrial applications. Mater. Charact..

[10] Hedrich S., Schlömann M., Johnson D.B. (2011). The iron-oxidizing proteobacteria. Microbiology.

[11] Xu X.W., Wu M., Oren A. (2014). The Family Kordiimonadaceae. The Prokaryotes.

[12] Tonon L.A.C., Moreira A.P.B., Thompson F. (2014). The Family Erythrobacteraceae. The Prokaryotes.

[13] Geszvain K., Butterfield C., Davis R., Madison A., Lee S., Parker D., Soldatova A., Spiro T.G., Luther G.W., Tebo B. (2012). The molecular biogeochemistry of manganese (II) oxidation. Biochem. Soc. Trans..

[14] Chen C.L., Maki J.S., Rittschof D., Teo S.L.M. (2013). Early marine bacterial biofilm on a copper-based antifouling paint. Int. Biodeter. Biodegrad..

[15] Holmström C., Egan S., Franks A., McCloy S., Kjelleberg S. (2002). Antifouling activities expressed by marine surface associated *Pseudoalteromonas* species. FEMS Microbiol. Ecol..

[16] Bernbom N., Ng Y.Y., Olsen S.M., Gram L. (2013). *Pseudoalteromonas* spp. Serve as Initial Bacterial Attractants in Mesocosms of Coastal Waters but Have Subsequent Antifouling Capacity in Mesocosms and when Embedded in Paint. Appl. Environ. Microbiol..

[17] Moradi M., Song Z., Yang L., Jiang J., He J. (2014). Effect of marine Pseudoalteromonas sp. on the microstructure and corrosion behaviour of 2205 duplex stainless steel. Corros. Sci.

[18] Liang R., Grizzle R.S., Duncan K.E., McInerney M.J., Suflita J.M. (2014). Roles of thermophilic thiosulfate-reducing bacteria and methanogenic archaea in the biocorrosion of oil pipelines. Front. Microb..

[19] Lenhart T.R., Duncan K.E., Beech I.B., Sunner J.A., Smith W., Bonifay V., Biri B., Suflita J.M. (2014). Identification and characterization of microbial biofilm communities associated with corroded oil pipeline surfaces. Biofouling.

[20] Zhang T., Fang H.H.P., Ko B.C.B. (2003). Methanogen population in a marine biofilm corrosive to mild steel. Appl. Microbiol. Biotechnol..

[21] Könneke M., Bernhard A.E., José R., Walker C.B., Waterbury J.B., Stahl D.A. (2005). Isolation of an autotrophic ammonia-oxidizing marine archaeon. Nature.

[22] Hügler M., Sievert S.M. (2011). Beyond the Calvin cycle: Autotrophic carbon fixation in the ocean. Mar. Sci..

[23] Lloyd K.G., Schreiber L., Petersen D.G., Kjeldsen K.U., Lever M.A., Steen A.D., Stepanauskas R., Richter M., Kleindienst S., Lenk S. (2013). Predominant archaea in marine sediments degrade detrital proteins. Nature.

[24] Qu Q., Wang L., Li L., He Y., Yang M., Ding Z. (2015). Effect of the fungus, *Aspergillus niger*, on the corrosion behaviour of AZ31B magnesium alloy in artificial seawater. Corros. Sci..

[25] Naranjo L., Pernía B., Inojosa Y., Rojas D., D’Anna L., González M., Sisto Á. (2015). First Evidence of Fungal Strains Isolated and Identified from Naphtha Storage Tanks and Transporting Pipelines in Venezuelan Oil Facilities. Adv. Microbiol..

[26] Miyata N., Tani Y., Maruo K., Tsuno H., Sakata M., Iwahori K. (2006). Manganese (IV) oxide production by Acremonium sp. strain KR21–2 and extracellular Mn (II) oxidase activity. Appl. Environ. Microbiol..

[27] Little B., Staehle R., Davis R. (2001). Fungal influenced corrosion of post-tensioned cables. Int. Biodeterior. Biodegrad..

[28] Pereira V.J., Marques R., Marques M., Benoliel M.J., Crespo M.B. (2013). Free chlorine inactivation of fungi in drinking water sources. Water Res..

[29] Jelic-Mrcelic G., Sliskovic M., Antolic B. (2006). Biofouling communities on test panels coated with TBT and TBT-free copper based antifouling paints. Biofouling.

[30] De Messano L.V., Sathler L., Reznik L.Y., Coutinho R. (2009). The effect of biofouling on localized corrosion of the stainless steels N08904 and UNS S32760. Int. Biodeterior. Biodegrad..

[31] Coetser S.E., Cloete T.E. (2005). Biofouling and biocorrosion in industrial water systems. Crit. Rev. Microbiol..

[32] Valster R.M., Wullings B.A., van der Kooij D. (2010). Detection of protozoan hosts for *Legionella pneumophila* in engineered water systems by using a biofilm batch test. Appl. Environ. Microbiol..

[33] Al-Ahmad M., Abdul Aleem F.A., Mutiri A., Ubaisy A. (2000). Biofouling in RO membrane systems. Part 1: Fundamentals and control. Desalination.

[34] Demadis K.D., Neofotistou E., Mavredaki E., Tsiknakis M., Sarigiannidou E.-M., Katarachia S.D. (2005). Inorganic foulants in membrane systems: Chemical control strategies and the contribution of “green chemistry”. Desalination.

[35] Euvrard M., Hadi L., Foissy A. (2007). Influence of PPCA (phosphinopolycarboxylic acid) and DETPMP (diethylenetriaminepentamethylenephosphonic acid) on silica fouling. Desalination.

[36] Hellio C., Yebra D. (2009). Advances in Marine Antifouling Coatings and Technologies.

[37] Al-Turaif H.A. (2013). Surface morphology and chemistry of epoxy-based coatings after exposure to ultraviolet radiation. Prog. Org. Coat..

[38] Daghia F., Zhang F., Cluzel C., Ladeveze P. (2015). Thermo-mechano-oxidative behaviour at the ply’s scale: The effect of oxidation on the transverse cracking in carbon-epoxy composites. Compos. Struct..

[39] Shams El Din A.M., El-Dahshan M.E., Tag El Din A.M. (2003). Biofilm formation on stainless steels Part 2. The role of seasonal changes, seawater composition and surface roughness. Desalination.

[40] Muyzer G., De Waal E., Uitterlinden A. (1993). Profiling of complex microbial populations by denaturing gradient gel electrophoresis analysis of polymerase chain reaction-amplified genes coding for 16S rRNA. Appl. Environ. Microbiol..

[41] Bano N., Ruffin S., Ransom B., Hollibaugh J.T. (2004). Phylogenetic composition of arctic ocean archaeal assemblages and comparison with antarctic assemblages. Appl. Environ. Microbiol..

[42] Barns S.M., Fundyga R.E., Jeffries M.W., Pace N.R. (1994). Remarkable archaeal diversity detected in a Yellowstone National Park hot spring environment. Proc. Natl. Acad. Sci. USA.

[43] Haugland R., Vesper S. (2002). Method of Identifying and Quantifying Specific Fungi and Bacteria. U.S. Patent.

[44] Klindworth A., Pruesse E., Schweer T., Peplies J., Quast C., Horn M., Glöckner F.O. (2012). Evaluation of general 16S ribosomal RNA gene PCR primers for classical and next-generation sequencing-based diversity studies. Nucl. Acids Res..

[45] Gardes M., Bruns T.D. (1993). ITS primers with enhanced specificity for basidiomycetes—Application to the identification of mycorrhizae and rusts. Mol. Ecol..

[46] Caporaso J.G., Kuczynski J., Stombaugh J., Bittinger K., Bushman F.D., Costello E.K., Fierer N., Gonzalez Pena A., Goodrich J.K., Gordon J.I. (2010). QIIME allows analysis of high-throughput community sequencing data. Nat. Methods.

[47] Edgar R.C. (2010). Search and clustering orders of magnitude faster than BLAST. Bioinformatics.

[48] DeSantis T.Z., Hugenholtz P., Larsen N., Rojas M., Brodie E.L., Keller K., Huber T., Dalevi D., Hu P., Andersen G.L. (2006). Greengenes, a chimera-checked 16S rRNA gene database and workbench compatible with ARB. Appl. Environ. Microbiol..

[49] Kõljalg U., Nilsson R.H., Abarenkov K., Tedersoo L., Taylor A.F.S., Bahram M., Bates S.T., Bruns T.D., Bengtsson-Palme J., Callaghan T.M. (2013). Towards a unified paradigm for sequence-based identification of Fungi. Mol. Ecol..

[50] Wang Q., Garrity G.M., Tiedje J.M., Cole J.R. (2007). Naïve Bayesian Classifier for Rapid Assignment of rRNA Sequences into the New Bacterial Taxonomy. Appl. Environ. Microbiol..

[51] Altschul S., Gish W., Miller W., Myers E., Lipman D. (1990). Basic local alignment search tool. J. Mol. Biol..

[52] Hammer Ø., Harper D.A.T., Ryan P.D. (2001). PAST: Paleontological statistics software package for education and data analysis. Palaeontol. Electron..

