# Supplementary figures and images for: Influence of Chlorination and Choice of Materials on Fouling in Cooling Water System under Brackish Seawater Conditions
Source: Materials (Basel). 2016 Jun 15;9(6):475. doi: 10.3390/ma9060475 (PMC5456818; doi:10.3390/ma9060475)

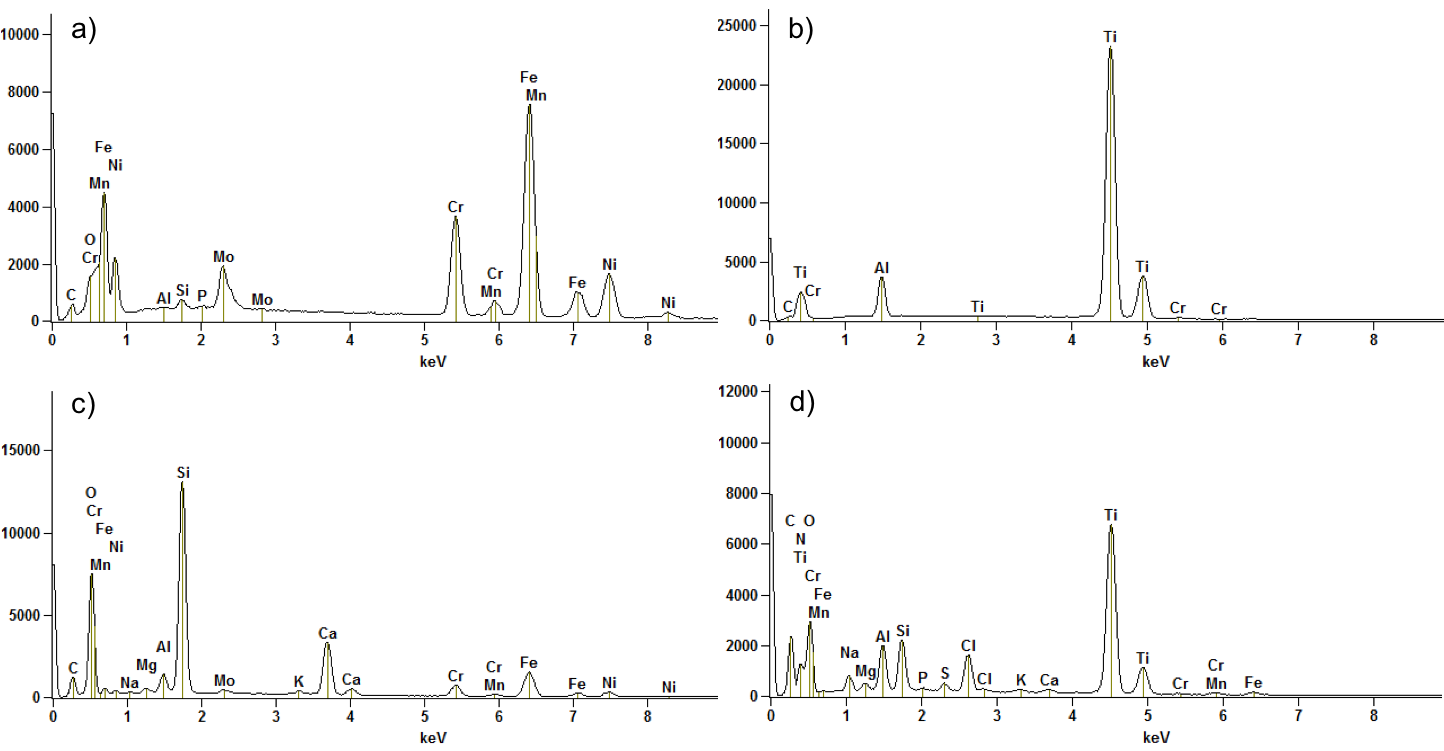

Supplement: Supplementary file 1 [file materials-09-00475-s001.zip › FigureS1.tiff]

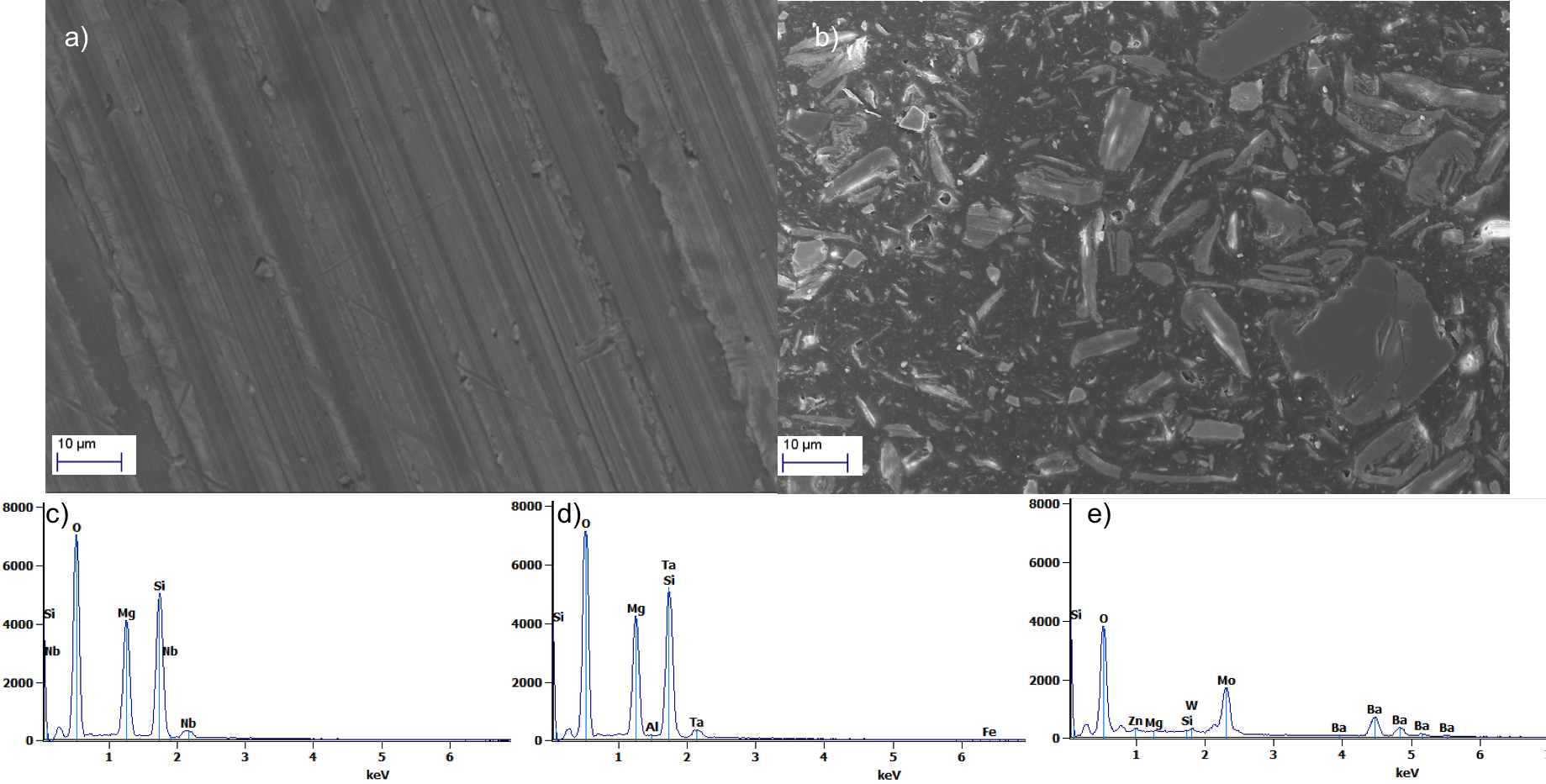

Supplement: Supplementary file 1 [file materials-09-00475-s001.zip › FigureS2.tiff]

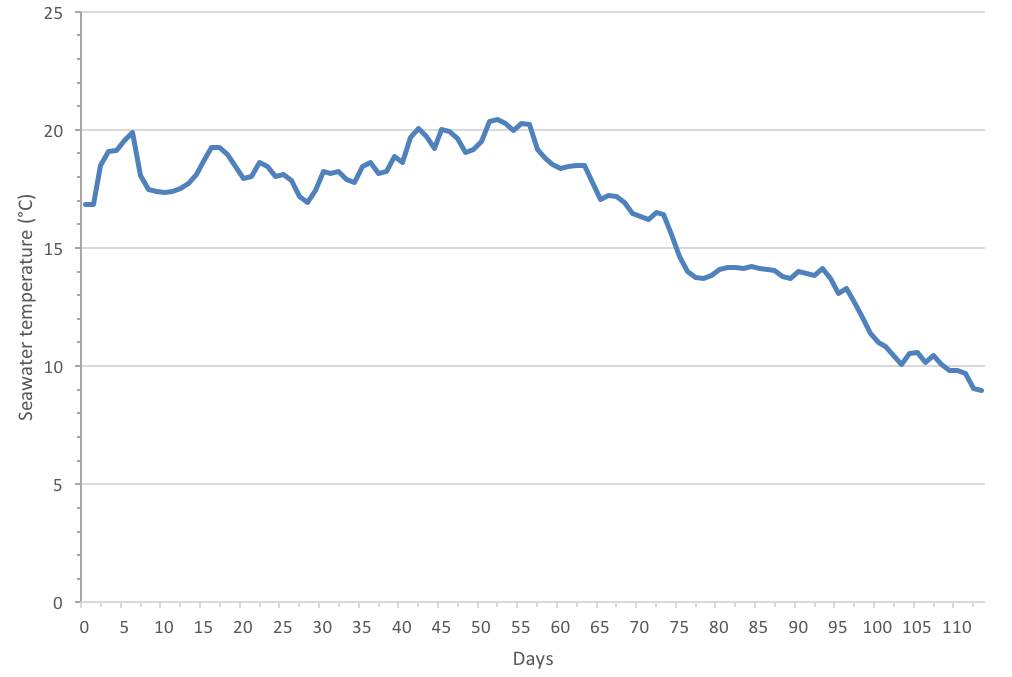

Supplement: Supplementary file 1 [file materials-09-00475-s001.zip › FigureS3.tiff]
